# Voice from both sides: a molecular dialogue between transcriptional activators and repressors in seed-to-seedling transition and crop adaptation

**DOI:** 10.3389/fpls.2024.1416216

**Published:** 2024-08-06

**Authors:** Dongeun Go, Bailan Lu, Milad Alizadeh, Sonia Gazzarrini, Liang Song

**Affiliations:** ^1^ Department of Botany, University of British Columbia, Vancouver, BC, Canada; ^2^ Department of Biological Science, University of Toronto Scarborough, Toronto, ON, Canada; ^3^ Department of Cell and Systems Biology, University of Toronto, Toronto, ON, Canada

**Keywords:** climate, crop adaptation, epigenetic regulation, HDAC, LAFL, PKL, PRC, Sdr4

## Abstract

High-quality seeds provide valuable nutrients to human society and ensure successful seedling establishment. During maturation, seeds accumulate storage compounds that are required to sustain seedling growth during germination. This review focuses on the epigenetic repression of the embryonic and seed maturation programs in seedlings. We begin with an extensive overview of mutants affecting these processes, illustrating the roles of core proteins and accessory components in the epigenetic machinery by comparing mutants at both phenotypic and molecular levels. We highlight how omics assays help uncover target-specific functional specialization and coordination among various epigenetic mechanisms. Furthermore, we provide an in-depth discussion on the Seed dormancy 4 (Sdr4) transcriptional corepressor family, comparing and contrasting their regulation of seed germination in the dicotyledonous species Arabidopsis and two monocotyledonous crops, rice and wheat. Finally, we compare the similarities in the activation and repression of the embryonic and seed maturation programs through a shared set of *cis*-regulatory elements and discuss the challenges in applying knowledge largely gained in model species to crops.

## Introduction

Seeds contributed greatly to the successful colonization of dry land by spermatophytes (seed plants) by allowing reproduction in the absence of water and dispersal under unfavorable growth conditions. During mid-to-late development, seeds enter the maturation phase, during which the seed accumulates storage compounds (lipids, proteins, or polysaccharides depending on the species) that are needed during germination to sustain post-germinative growth. Seeds can contribute up to 70% of our caloric intake, as food and livestock feed, and therefore play a fundamental role in human nutrition. For these reasons, there is a great interest in understanding the gene regulatory networks (GRNs) controlling seed development and germination to improve qualitative and quantitative traits associated with these processes.

GRN depicts transcriptional regulators and their target genes as nodes and the regulatory relationships as directed edges (Barabási and Oltvai, 2004). GRN analysis often employs one or more of model-, information theory-, and machine learning-based methods (Zhao et al., 2021). Although barebone GRNs can be inferred from transcription profiles of transcription factors (TFs) and their potential target genes based on co-expression in bulk tissues, higher resolution of transcriptome profiles and data examining additional regulatory features will provide valuable information for more accurate inference and a higher understanding of context-specific regulation. However, data availability differs by biological systems. In animals, single-cell (sc) omics data are widely available. These datasets are increasingly paired, which means various types of omics data are collected from the same cells. Equally importantly, a plethora of tools, developed and validated with multi-omics data in animals, are available to infer GRNs (Badia-i-Mompel et al., 2023). By contrast, although a growing amount of plant sc transcriptome and ATAC-seq profiles became available in recent years, sc datasets are still relatively scarce in number and type in plants, and multi-omics assays are usually generated from separate samples, resulting in reduced data resolution, increased noise, and fewer features for multimodal predictions of important biological processes. To date, plant sc transcriptome profiles of vegetative tissues have provided excellent insights into plant development (Shahan et al., 2022), evolution (Guillotin et al., 2023), and response to environmental cues (Wendrich et al., 2020). TF binding sites are widely used together with sc profiles of transcriptome or chromatin accessibility in integrative analysis pipelines such as MINI-EX and MINI-AC to infer cell type-specific GRN (Ferrari et al., 2022; Manosalva Pérez et al., 2024).

There are several challenges in studying transcriptional repression in seeds. First, high-resolution data are limited, especially during late seed maturation. Second, the regulation of gene repression is arguably more complex. For instance, the inference of gene activation is relatively straightforward based on open chromatin, binding (sites) of transcriptional activators, and elevated transcript abundance. By contrast, lack of transcription could result from either active repression or a lack of activation, which may not be distinguishable solely by chromatin accessibility assays. Additionally, both computational analysis (Brooks et al., 2021) and experimental evidence (Zhu et al., 2024) show that TFs may play a dual role of activation and repression depending on their interacting partners, and such characterizations are relatively limited in seeds. In this review, we highlight various omics datasets useful for GRN inference in seeds. We place a special emphasis on the repressive machinery by detailing the genetic and functional genomic characterizations to provide context for their mode of action. Considering complex biological networks are hierarchical and scale-free (Basso et al., 2005), we focus on the master LAFL TFs in the examples because these hubs are likely to capture extensive regulation.

The function of LAFL in seed development and maturation has been extensively studied in Arabidopsis (Lepiniec et al., 2018; Alizadeh et al., 2021; Gazzarrini and Song, 2024). *LEC1, FUS3*, and *LEC2* transcripts accumulate since the zygote or pre-globular stage of embryogenesis, and the expression of ABI3 initiates later at the globular stage. These TFs play a prominent role during seed maturation, where they are required for the accumulation of seed storage compounds, such as triacylglycerols (TAGs), seed storage proteins (SSPs), oleosins (OLEO), and stress proteins (LEA) (Giraudat et al., 1992; Meinke, 1992; Meinke et al., 1994; West et al., 1994; Roscoe et al., 2015). LAFL also promote seed dormancy and suppress precocious germination of immature seeds, known as vivipary or pre-harvest sprouting (PHS) in cereals, by inhibiting cell division, the activity of the shoot and root meristems, and the differentiation of the cotyledon epidermis (trichome development) and vascular system (xylem) (Meinke, 1992; Keith et al., 1994; Nambara et al., 1995; Raz et al., 2001; Bryant et al., 2019). LAFL’s functions in seed development and germination are partly mediated by the hormones auxin, ABA, GA, ethylene, brassinosteroids, and jasmonate (Parcy et al., 1994; Curaba et al., 2004; Gazzarrini et al., 2004; Braybrook et al., 2006; Stone et al., 2008; Lumba et al., 2012; Ryu et al., 2014; Chiu et al., 2016; Pan et al., 2020). The spatiotemporal expression patterns of *LAFL* are tightly controlled at the transcriptional and epigenetic levels. Epigenetic silencing of *LAFL* is required to promote post-embryonic development (Lepiniec et al., 2018; Alizadeh et al., 2021; Gazzarrini and Song, 2024).

Seed quality is a holistic term that includes seed viability, moisture and nutrient content, depth of dormancy, longevity, and vigor. Some of these traits, such as seed vigor, are assessed during the transition from seed to seedlings (Finch-Savage and Bassel, 2016). Therefore, we focus on the regulation and performance of seed-to-seedling transition by using this process as a proxy of seed quality in this review. Recent advances made possible by omics tools provide a rich resource to compare and contrast various epigenetic machinery that regulate the transition from seed to seedlings. The information will also provide mechanistic context for GRN inference at this developmental stage. Using a novel transcriptional cofactor, Seed dormancy 4 (Sdr4), as an example, we summarize and discuss the role of this corepressor and its orthologs in Arabidopsis, rice, and wheat. We also discuss cis-regulatory elements (CREs) important for seed maturations as well as a potential regulatory symmetry exerted on these CREs, which together facilitate the activation and repression of the seed maturation program.

## Phenotypic and omics resemblance of genetic and epigenetic regulators

### Functional characterization of players in seed-to-seedling transition by phenotypic resemblance and genetic evidence

**Figure 1:**
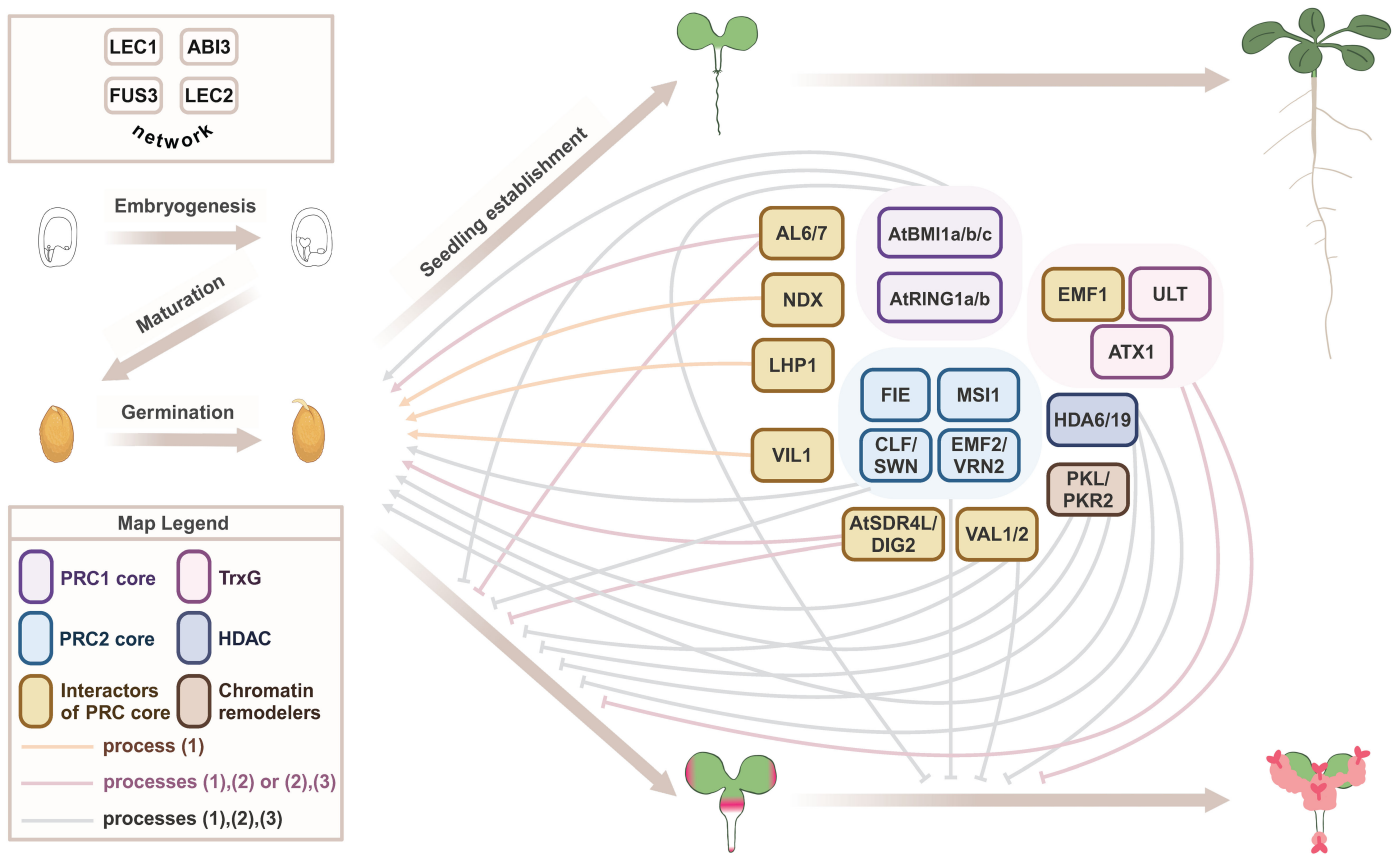
Epigenetic regulators that repress the seed maturation program during seed-to-seedling transition. A proposed model on the regulation of early developmental phase transitions by general and stage-specific epigenetic machinery in Arabidopsis (**Table 1**). Embryogenesis and maturation phases of seed development are regulated by the LAFL network (top left). The seed-to-seedling phase transition comprises the break of dormancy, germination, and seedling establishment. This normal developmental trajectory requires repression of the seed maturation program by a suite of epigenetic regulators. Loss-of-function mutations in these epigenetic regulators often lead to an alternative developmental trajectory, with mutant seeds exhibiting defects in one or more processes: (1) germination, (2) repression of post-embryonic accumulation of storage compounds, with pink color indicating the accumulation of storage lipids, and (3) repression of subsequent formation of callus and somatic embryos. Severe mutants of general epigenetic machinery usually show all three types of defects, whereas the role of stage-specific regulators may be limited to one or two processes.

The importance of shutting down the embryonic program and silencing *LAFL* at the end of seed development is clearly shown by *LAFL* overexpression/ectopic expression (OE) phenotypes, which include delayed germination, growth and flowering (*ABI3, FUS3*, and *LEC2*), development of cotyledon-like organs (*LEC1*, *FUS3*, and *LEC2*), somatic embryos, and callus-like structures (*LEC1* and *LEC2*) (**Table 1**). Ectopic expression of early-acting genes (*LEC1* and *LEC2*) shows the most dramatic phenotypes, such as somatic embryos and development of callus-like structures. These embryonic structures accumulate seed storage lipids (TAGs), and proteins (2S and 12S), as a result of ectopic expression of the seed maturation program (**Table 1**). Robust repression of *LAFL* and the embryonic program during vegetative development is orchestrated by a suite of epigenetic regulators, including Polycomb group (PcG) repressive complex 1 (PRC1) and PRC2, Trithorax group (TrxG), chromatin remodeling factors and other repressive proteins (**Figure 1**) (Xiao et al., 2017a; Lepiniec et al., 2018; Gazzarrini and Song, 2024). PRC1 catalyzes monoubiquitylation of lysine (K) 121 on histone 2A (H2AK121ub). PRC1 core components include BMI1A, BMI1B, BMI1C, RING1A, and RING1B (**Figure 1**), all of which have E3 ligase activity (Mozgova and Henning, 2015; Baile et al., 2022). PRC2 promotes trimethylation of H3 on K27 (H3K27me3), catalyzed by the histone methyltransferases SWN, CLF, and MEA. Arabidopsis has three core PRC2 complexes: EMF-PRC2, composed of EMF2, CLF/SWN, FIE, and MSI1; VRN-PRC2, composed of VRN2, CLF/SWN, FIE, and MSI1; and FIS-PRC2, which includes FIS2, MEA, FIE, and MSI1 (**Figure 1**). These PRC2 complexes have overlapping as well as specific functions throughout development (Bieluszewski et al., 2021; Baile et al., 2022). Several PRC1 and PRC2 accessory proteins that physically interact with the PRC core components to repress transcription, such as VAL1/2, HDAC, LHP1/TFL2, AL6, AL7, EMF1, NDX, and VRN5/VIL1, as well as TrxG factors such as H3K4me3 methyltransferases ATX and ULT1, chromatin remodeling factors such as PKL and PKR2, and corepressors such as TPL, TPR, and AtSDR4L, aid in repression of *LAFL* and the embryonic program during vegetative development (Gazzarrini and Song, 2024).

**Table 1:** Genetic and epigenetic regulators in seed-to-seedling transition.

OE/mutant	Phenotypes	Genetic and genomic insights related to seeds and seedlings
*LEC1* OE	**Storage compounds:** Ectopic lipids and starch in *lec1-d ^tnp^* (Casson and Lindsey, 2006). Ectopic cruciferin and starch in *35S:LEC1-GR* seedlings induced 0–3 days after imbibition (DAI) (Junker et al., 2012). **Callus and somatic embryos (SE):** Cotyledon-like organs and somatic embryos in *35S:LEC1* seedlings (Lotan et al., 1998). Swollen hypocotyl, but no embryonic callus in *lec1-d ^tnp^* (dominant mutant). Phenotype enhanced by auxin, sugar or GA inhibitor (paclobutrazol), but not rescued by GA (Casson and Lindsey, 2006). *35S:LEC1-GR* seedlings show different phenotypes dependent on time of induction: most show cotyledon-like organs, swollen and green roots, and callus and somatic embryos, and 10%–40% show arrested roots (0–2 DAI, days after imbibition); long hypocotyls with an apical hook (3 DAI); no phenotype if induced at 4 DAI, but induction at 4DAI+ABA resulted in cotyledon-like leaves that express *CRU* (Junker et al., 2012).	**Selected marker genes:** Ectopic expression (*in situ* hyb) of seed storage proteins: 12S cruciferins (*CRA*), S3 oleosin (*OLEO*) in *35S:LEC1* seedlings (Lotan et al., 1998). Ectopic expression of *LEC2, FUS3, ABI3*, and maturation genes (*2S* albumin*, CRC*) in *35S:LEC1-GR* seedlings (Kagaya et al., 2005b). Ectopic expression of *CRU, LEC2, FUS3*, and *ABI3* in *35S:LEC1-GR* seedlings induced 0–3 DAI (Junker et al., 2012) (Junker et al., 2012). *35S:LEC1-GR* associates with *LAFL*, *WRI*, and seed maturation genes in ChIP-chip in seedlings (Pelletier et al., 2017). **Omics datasets:** GSE22352 (ChIP-chip of LEC1 of 2-week-old *35S:LEC1:GR* seedlings treated by DEX or mock for 24 h), GSE22173 (ATH1 microarray of 2-week-old *35S:LEC1-GR* treated by DEX or mock for 8 h with and without ABA) (Junker et al., 2012). GSE99528 (microarray of 8-day-old *35S:LEC1:GR* treated with and without DEX for 1 h), GSE99529 (ChIP-chip of LEC1 in 8-day-old *35S:LEC1-GR* seedlings either grown on DEX plates or treated by DEX for 4 h), GSE99587 (ChIP-seq of LEC1 in *LEC1:LEC1-GFP: LEC1*/*lec1–1* seeds at the bent cotyledon-stage) (Pelletier et al., 2017).
*LEC2* OE	**Storage compounds:** Ectopic lipids and starch accumulation in *35S:LEC2-GR* ovules (Stone et al., 2008). Ectopic accumulation of seed-specific lipids and triacylglycerol (TAGs) in leaves of 35S:LEC2-GR induced after 2 weeks (Santos Mendoza et al., 2005) **Callus and SE:** Somatic embryos in *35S:LEC2* (Stone et al., 2001).	**Selected marker genes:** Ectopic expression of *CRA* (*12S*) and *OLEO* detected by *in situ* hybridization in *35S:LEC2* seedlings (Stone et al., 2001). Ectopic expression (RT-PCR) of *LEC1*, *FUS3*, *2S, CRA1*, and *OLEO* in *35S:LEC2-GR* seedlings (Stone et al., 2001). Ectopic expression of *S3* (*OLEO*), *2S3* (albumin), and *LAFL* in leaves of *35S:LEC2-GR* (Santos Mendoza et al., 2005). Ectopic expression (microarray and/or RT-PCR) of *LEC1*, *FUS3*, *2S*, *CRA1*, and *OLEO* in *35S:LEC2-GR*. LEC2 associates (ChIP) with *2S3* and *OLEO* in *35S:LEC2-GR* seedlings (Stone et al., 2008). **Omics datasets:** GSE3959 (ATH1 microarray of 8-day-old *35S:LEC2-GR* seedlings treated by DEX for 1 and 4 h) (Braybrook et al., 2006).
*FUS3* OE	**Dormancy and germination:** Delayed germination, vegetative growth, and flowering of *fus3 ML1:FUS3*. Strong lines are arrested at the seedling stage (Gazzarrini et al., 2004; Tsai and Gazzarrini, 2012). *ML1:FUS3* seeds hypersensitive to ABA, sorbitol, and glucose during germination (Tsai and Gazzarrini, 2012). **Storage compounds:** Ectopic accumulation of seed storage proteins (2S, 12S) in *ML1:FUS3* leaves (Gazzarrini et al., 2004). Increased TAGs content in estradiol-inducible *XVEpro: FUS3* (Zhang et al., 2016). **Callus and SE:** Development of cotyledon-like organs, arrested seedlings, but no somatic embryos in *fus3 ML1:FUS3* strong lines. Delayed flowering and cotyledon-like leaves are partially rescued by GA (Gazzarrini et al., 2004).	**Selected marker genes:** Ectopic expression of *2S3* (enhanced by *ABA*). Ectopic expression of *CRC* only in +ABA in 7 DAI seedlings of DEX inducible *GRpro:FUS3* (Kagaya et al., 2005a). Repression of GA biosynthesis genes in *fus3 ML1:FUS3* seedlings (*GA20ox* and *GA3ox*) (Gazzarrini et al., 2004). Ectopic expression of *OLEO*, *2S3*, *CRU*, and *WRI* in *ESTpro: FUS3* (Zhang et al., 2016). **Genomics datasets:** GSE43291 (ChIP-chip of *FUS3:FUS3-myc*/*fus3–3* embryonic culture) (Wang and Perry, 2013) GSE80360 (ATH1 microarray of 8 day-old *XVEpro: FUS3* seedlings ± sucrose) (Zhang et al., 2016).
*ABI3* OE	**Dormancy and germination:** Increased sensitivity to ABA during seed germination (Zhang et al., 2005). Increased sensitivity to ABA in inhibition of root elongation (Parcy et al., 1994). **Storage compounds:** Increased TAGs content in *XVEpro: ABI3* (Yang et al., 2022b).	**Selected marker genes:** Ectopic expression of seed maturation genes *2S3, Em1, Em6*, and *CRC* in *35S:ABI3* in response to ABA (Parcy et al., 1994). Ectopic expression of *2S3*, and ectopic expression of *Em* and *CRC* in response to ABA in 7-day-old seedlings of *GRpro: ABI3* (Kagaya et al., 2005a). Ectopic depression of *FUS3*, *OLEO*, and *WRI* in *XVEpro: ABI3* (Yang et al., 2022b). **Omics datasets:** GSE150561 (microarray of wild-type and *abi3–5* seeds harvested at 15–16 days after flowering with and without placement on moist blotter for 1 day; ChIP-chip of *ABI3:ABI3-myc*/*abi3–5* embryonic culture) (Tian et al., 2020) PRJNA678646 (RNA-Seq of XVEpro: *ABI3* using fifth to eighth rosette leaves treated with β-estradiol or mock for 4 days) (Yang et al., 2022b).
*atbmi1a atbmi1b*	**Dormancy and germination:** Delayed germination both in unstressed condition and under salt or osmotic stress (Molitor et al., 2014) **Storage compounds:** Low penetrance of the pickle-root trait (~8%) (Chen et al., 2010a). **Callus and SE:** Low penetrance of embryonic callus (~18%) (Chen et al., 2010a). The same double mutant exhibits high penetrance (>75%) of callus and somatic embryos in another study (Bratzel et al., 2010).	**Selected marker genes:** Increased expression of *STM*, *WOX5*, *WUS*, *LEC1*, and *FUS3* (Bratzel et al., 2010). Derepression of *LAFL* in *atbmi1a atbmi1b* examined by RT-qPCR in 2-week-old seedlings (Chen et al., 2010a). Reduced H2AK121ub of *LEC1*, *FUS3*, *ABI3*, *WUS*, and *BBM* (Yang et al., 2013). Increased expression of *ABI3, DOG1, CRU1, CUR3, PER1*, and *CHO1*. Altered histone marks (H3K4me3, H3K27me3) (Molitor et al., 2014). **Omics datasets:** GSE67322 (RNA-seq and H3K27me3 ChIP-seq of 2-week-old *atbmi1a atbmi1b*, *atring1a atring1b*, *lhp1*, *clf*, and *clf swn* seedlings) showed that H3K27me3 and differential expression of seed maturation genes are similarity regulated in *clf swn*, *atring1a atring1b*, and *atbmi1a atbmi1b* (Wang et al., 2016). GSE89358 (RNA-seq of 7-day-old *atbmi1abc* and wild-type seedlings; H3K27me3 and H2AK121ub ChIP-seq of *atbmi1abc*, *clf swn*, *lhp1*, and wild-type seedlings) (Zhou et al., 2017). GSE83568 (RNA-seq of 10-day-old single, double, and triple mutants of *atbmi* and wild-type seedlings). PRJE 52473 (Hi-C and ChIP-seq of BMI1B-FLAG and H3K4me3 using 10-day-old seedlings) (Yin et al., 2023)
*atring1a atring1b*	**Dormancy and germination:** Hypersensitive to ABA in seedling establishment (Zhu et al., 2020). **Storage compounds:** moderate penetrance of the pickle-root phenotype (~50%) (Chen et al., 2010a). **Callus and SE:** Low penetrance of embryonic callus (~17%). Embryonic and pickle-root traits are attenuated by auxin transport inhibitor NPA (Chen et al., 2010a).	**Selected marker genes:** RT-qPCR showed that *LAFL* are derepressed in *atring1a atring1b* 2-week-old and 1-month-old seedlings (Chen et al., 2010a). *ABI3* is upregulated in *atring1a atring1b* (Zhu et al., 2020). **Omics datasets:** GSE67322 (see description in the *atbmi1a atbmi1b* row in this table). GSE155378 (ATAC-seq of *atbmi1abc*, *atring1a atring1b*, *clf swn*, *emf1*, and *lhp1*; H2AK121ub ChIP-seq of *emf1* and *atring1a atring1b*; H3K27me3 ChIP-seq of *atring1a atring1b* and *lhp1*; RNA-seq of *clf swn*, *atring1a atring1b* and *emf1*; all assays were carried out using 10-day-old whole seedlings and include wild-type Col-0 controls) (Yin et al., 2021).
*atring1a atring1b clf*	**Storage compounds:** *clf* slightly exacerbates the pickle-root phenotype of *atring1a atring1b* (Chen et al., 2010a). **Callus and SE:** *clf* substantially exacerbates the embryonic callus phenotype of *atring1a atring1b* (Chen et al., 2010a).	**Selected marker genes:** RT-PCR showed upregulation of *LEC1, LEC2*, and *FUS3* is further increased in *atring1a atring1b clf* compared to *atring1a atring1b* (Chen et al., 2010a).
*al6 al7*	**Dormancy and germination:** Delayed germination; enhanced under salt (NaCl) or osmotic (mannitol) stress (Molitor et al., 2014). **Storage compounds:** Increased level of *CRU1* and *CRU3* (3 DAI). Tissue-level defects not observed.	**Selected marker genes:** Increased expression of *ABI3, DOG1, CRU1, CUR3, PER1*, and *CHO1*, but much lower than in *atbmi1a atbmi1b*. Altered histone marks at *ABI3* and *DOG1* in 3 DAG (increased H3K4m3, decreased H3K27me3) albeit less than in *atbmi1a atbmi1b*. AL6 shows similar binding to LHP1 at the *ABI3* and *DOG1* loci (Molitor et al., 2014)
*atbmi1a atbmi1b al6 al7*	**Dormancy and germination:** Further delayed germination on regular plates and under salt (NaCl) or osmotic (mannitol) compared to *atbmi1a atbmi1b* and *al6 al7* double mutants (Molitor et al., 2014).	
*ndx*	**Dormancy and germination:** Hypersensitive to ABA during seedling establishment (greening) and root growth (Zhu et al., 2020).	**Selected marker genes:** *ABI4* and *ABI5* upregulation in *ndx*, and *ABI3* and *ABI4* upregulation in *ndx* + ABA; RNA-seq and RT-qPCR showed that NDX binds to *ABI3*, *ABI4*, and *ABI5*, but associates only with *ABI4* in ChIP-qPCR. Upregulation of these genes is much stronger in *atring1a atring1b.* ChIP-qPCR showed that levels of H2AK121ub are reduced at *ABI4, Em1*, and *SUT4*, and slightly at *ABI3* in *ndx-5* and to a similar level to *atring1a atring1b* (Zhu et al., 2020). **Omics datasets:** PRJNA556351 (RNA-seq of 7-day-old seedlings *ndx*, *atring1a atring1b*, and Col-0 ± ABA) (Zhu et al., 2020). GSE201841 (RNA-seq, BS-seq, and sRNA-seq DRIP-seq of *ndx1–4* and wild-type control. ChIP-seq of flag-NDX and NDX-GFP. Ten-day-old seedlings were used for the sequencing assays) (Karányi et al., 2022).
*lhp1* (*tfl2*)	**Dormancy and germination:** Elevated expression of *DOG1*. Delayed germination on ABA plates, possibly mediated by ANAC019 and ANAC055 (Ramirez-Prado et al., 2019).	**Selected marker genes:** LHP1 binds to *ABI3* and *DOG1* (Molitor et al., 2014). Modest upregulation of *DOG1* in *lhp1* mutant (Chen et al., 2020). No deregulation of *FUS3* or *ABI3* in 14-day-old *lhp1* seedlings (Ramirez-Prado et al., 2019). **Omics datasets:** DamID-chip, an *E. coli* Dam fused with LHP1 followed by tiling microarray profiling, and A-MEXP-602 (chip-chip of LHP1 and H3K27me3 in 10-day-old wild-type and *lhp1* seedlings) showed that LHP1 colocalizes genome-wide with H3K27me3 (Turck et al., 2007; Zhang et al., 2007). GSE76571 (ChIP-seq of LHP1 and H3K27me3 in 14-day-old wild-type, *lhp1*, and *clf* seedlings) showed LHP1 is involved in the spreading of H3K27me3 and shaping chromatin topology (Veluchamy et al., 2016). GSE67322 (see description in the *atbmi1a atbmi1b* row in this table). GSE89358 (see description in the *atbmi1a atbmi1b* row in this table). GSE155378 (see description in the *atring1a atring1b* row in this table).
*emf1*	**Callus and SE:** *emf1–2* forms oval-shaped, petiole-less cotyledons that develop into carpeloid, and no leaf primordia and do not produce vegetative rosettes (Sung et al., 1992; Chen et al., 1997). A small % of *emf1–2* and *emf1–2 emf2–1* plants form callus after 1 month of culture (Calonje et al., 2008).	**Selected marker genes:** Increased *ABI3* and *At2S3* and decreased *LEC1* and *LEC2* transcript abundance in 14-day-old seedlings (Xu et al., 2018). **Genomics datasets:** GSE155378 (see description in the *atring1a atring1b* row in this table).
*emf1 atx*, *emf1 ult*, *emf1 atx1 ult1*	**Storage compounds:** Increased storage lipids in pickle-root regions of *emf1 atx, emf1 ult*, and *emf1 atx ult* (Xu et al., 2018). **Callus and SE:** *emf1 atx (4%), emf1 ult (11%)*, and *emf1 atx ult (22%)* show embryo- and callus-like structures arising from cotyledons, hypocotyls, and roots; roots are arrested and swollen, pkl-like. The pickle-root phenotype is enhanced by a GA biosynthesis inhibitor, PAC. *atx*, *ult*, or *atx ult* does not show any of these phenotypes (Xu et al., 2018).	**Selected marker genes:** Upregulation of *ABI3*, *FUS3*, *LEC2*, seed maturation genes (*2S*, *OLEO, CRU*, and *LEA*), dormancy (*DOG1*), GA catabolism (*GA2ox1*) and GA signaling repressor (*RGL1*), and downregulation of GA synthesis (*GA3ox1*) in 14-day-old *emf1 atx1 ult1* seedlings by qRT-PCR and/or RNA-seq (*ABI3*, *FUS3*, *LEC2*, and 2S3 are also upregulated in *emf1 ult* but not in *atx ult*, by qRT-PRC). *LEC1* is downregulated in *atx, ult, emf1*, *atx ult*, and *ult atx emf*. ChIP-seq and ChIP-qPCR showed that ULT and ATX are associated with *ABI3*, *LEC2*, and *2S3*, by ChIP-seq and ChIP-qPCR. Decreased H3K27me3 marks at *ABI3*, *LEC2*, seed maturation genes (*CRU* and *OLEO*) and dormancy (*DOG1*) in *emf1 atx1 ult1* (Xu et al., 2018).
*fie*	**Dormancy and germination:** Delayed germination and 40% dormant seeds, delayed cotyledon greening similar to WT germinated on ABA; these phenotypes are not rescued by GA (Bouyer et al., 2011). **Storage compounds:** Sugar-enhanceable accumulation of storage reserves at the root tip and in the aerial part (Bouyer et al., 2011). **Callus and SE:** Development of somatic embryos and callus-like structures in seedlings (Makarevich et al., 2006; Bouyer et al., 2011).	**Selected marker genes:** Strong decrease in H3K27me3 levels and *ABI3, FUS3*, and *LEC2* derepression. Upregulation of seed maturation (*CRU3*, *CRA1*, *2S1*, *2S2*, *OLEO*, and *LEA*), dormancy (*DOG1* and *SOM*), and ABA signaling (*ABI4*) (Bouyer et al., 2011). **Omics datasets:** GPL10918 (ChIP-chip H3K27me3 and H3K4me3 in 20-day-old *fie* and wild-type seedlings) (Bouyer et al., 2011). GSE95562 (ChIP-seq of FIE in 30-h-old *pRNAi-BPC*; *pRNAi-ZnF* double knockdown mutant and wild-type Ws accession); GSE84483 (ChIP-seq of pFIE: FIE-HA in 30-h-old *fie-11* in C24 accession) (Xiao et al., 2017b).
*clf*	**Dormancy and germination:** Two mutant alleles of *clf* show reduced seed yield and increased cell size and seed size (Liu et al., 2016). **Storage compounds:** Increased level of FA and oil.	**Selected marker genes:** Upregulation of *LAFL*, several *OLE*, and *WRI* in *clf* siliques; upregulation of *FUS3* and *ABI3* and downregulation of *LEC1* in mat green *clf* embryo. *clf* seedlings: decreased H3K27me3 at *FUS3*, *ABI3* (Liu et al., 2016). **Omics datasets:** GSE7065 (ChIP-chip of 10- to 14-day-old 35S::GFP-CLF in *clf-50* in Ws accession). GSE67322 (see description in the *atbmi1a atbmi1b* row in this table). GSE103361 (RNA-seq and H3K27me3 ChIP-seq of 3-week-old shoots of *pkl*, *clf*, and wild-type plants) (Carter et al., 2018). GSE155502 (HiChIP of H3K27me3 and H3K9ac and Hi-C in 14-day-old wild-type and *clf* seedlings) showed altered H3K27me3 repressive loops in *clf* (Huang et al., 2021).
*swn clf*	**Dormancy and germination:** Delayed germination, stronger than *fie* (Bouyer et al., 2011). **Storage compounds:** Increase accumulation of storage reserves. **Callus and SE:** Development of somatic embryos and callus-like structures in seedlings. Swollen root that produced green shoot-like tissue, similar to *fie* (Chanvivattana et al., 2004; Makarevich et al., 2006).	**Selected marker genes:** *LEC1, LEC2*, and *FUS3* derepression in seedlings; strong *FUS3* (not *LEC1* or *LEC2*) upregulation in 3DAP siliques (Makarevich et al., 2006). **Omics datasets:** GSE67322 (see description in the *atbmi1a atbmi1b* row in this table). GSE89358 (see description in the *atbmi1a atbmi1b* row in this table). GSE98301 (RNA-seq of 10-day-old *clf-50 swn-1* and wild-type seedlings treated with ABA for 5 h or 4 days. RNA-seq of 10-day-old *clf-50*, *swn-1*, *clf-50 swn-1*, and wild-type seedlings mock-treated for 4 days) (Liu et al., 2019). GSE108960 (RNA-seq of 10-day-old *clf*, *swn*, *clf swn*, and wild-type seedlings, and ChIP-seq of 10-day-old CLF-GFP and SWN-GFP seedlings) showed CLF and SWN function redundantly to deposit H3K27me3 at *LAFL* loci (Shu et al., 2019). GSE155378 (see description in the *atring1a atring1b* row in this table).
*swn clf pkl*	**Storage compounds:** Accumulation of storage lipids detected by Fat Red B staining (Aichinger et al., 2009). **Callus and SE:** *pkl* enhances embryo and callus-like structure compared to *swn clf* (Aichinger et al., 2009).	**Selected marker genes:** *LEC1* and *FUS3* are synergistically upregulated in *pkl clf* double mutant (Aichinger et al., 2009).
*emf2 vrn2*	**Storage compounds:** Ectopic storage lipid and chlorophyll accumulation in *emf2 vrn2* seedling roots (Ikeuchi et al., 2015). **Callus and SE:** Somatic embryos in *emf2 vrn2* (Schubert et al., 2005). Ectopic shoot on *emf2 vrn2* root (Ikeuchi et al., 2015).	**Selected marker genes:** Ectopic expression of *LEC1*, *LEC2*, and *FUS3* in *emf vrn2* roots (Ikeuchi et al., 2015).
*emf2 sdg8*	**Storage compounds:** Accumulation of storage proteins and lipids (Tang et al., 2012). **Callus and SE:** No somatic embryos in *emf2*, *vrn2*, or *sdg8*. Somatic embryos in *emf2 sdg8* (Tang et al., 2012).	**Selected marker genes:** Derepression of *FUS3*, seed maturation genes (*2S*, *LEA*, and *LTP*), and GA deactivation genes (*GA2ox*) in *sdg8* (Tang et al., 2012). Depression of *LAFL*, seed maturation genes (*2S*, *LEA*, and *LTP*), and GA deactivation genes (*GA2ox*) in *sdg8 emf2* (Tang et al., 2012).
*bpc1 bpc2*	**Dormancy and germination:** No dormancy and germination phenotype. Instead, double mutant is pleiotropic, exhibiting ovule and seed abortion, dwarfism, and reduced lateral roots (Monfared et al., 2011).	**Selected marker genes:** BPCs repress *LEC2* (Xiao et al., 2017b), *FUS3* (Xiao et al., 2017b; Wu et al., 2020), and *ABI4* (Mu et al., 2017). BPCs activate *LEC2* (Berger et al., 2011). **Omics datasets:** GSE84483 (ChIP-seq of 30-h-old gBPC1-Myc in Col-0 accession) (Xiao et al., 2017b).
*val1* (*hsi2*)	**Dormancy and germination:** Similar to WT (Chen et al., 2020). **Storage compounds:** Embryonic phenotypes such as cotyledon-like organs and ectopic embryos on leaves displayed by 23% of seedlings treated by the GA-biosynthesis inhibitor, paclobutrazol (Suzuki et al., 2007). **Callus and SE:** A small % of callus observed at cotyledon margin (only in *val1–1* in the WS background) (Suzuki et al., 2007).	**Selected marker genes:** Derepression of *CRC* and *2S2* in seedlings, and derepression of *LAFL*, *L1L*, *CRC*, and *2S1* in seedlings rescued from embryos 9 days after pollination (Suzuki et al., 2007; Jia et al., 2013). Depression of *LEC1*, *FUS3*, *ABI3*, *AGL15*, and *DOG1*, and reduced H3K27me3 level at *LEC1*, *ABI3*, *AGL15*, and *DOG1* loci in *hsi2–2*. ChIP-PCR showed that *AGL15* and *DOG1* are direct targets of HSI2 (Veerappan et al., 2012, 2014; Chen et al., 2018, 2020). **Omics datasets:** ATH1 microarray from 5-day-old seedlings of Col-0, *val1–2* and *val2–1*, and 7.5-day-old seedlings of *val1–2 val2–1* double mutants) (Suzuki et al., 2007). GSE74692 (RNA-seq time course of *val1* and wild-type developing seeds) (Schneider et al., 2016).
*val1 val2* (*hsi2 hsl1*)	**Dormancy and germination:** Reduced and delayed germination (Suzuki et al., 2007; Chen et al., 2020). **Storage compounds:** Over-accumulation of storage reserves in seedlings (Suzuki et al., 2007; Tsukagoshi et al., 2007). **Callus and SE:** Embryonic callus found both in shoot and root (Suzuki et al., 2007). Prevalent formation of callus if developing seeds of *val1 val2* are rescued on MS plates (Jia et al., 2013).	**Selected marker genes:** Stronger derepression of *LAFL*, *L1L*, and seed storage genes in *val1 val2* seedlings grown from mature seeds or rescued embryos than in *val1* seedlings (Suzuki et al., 2007; Jia et al., 2013). Derepression of *LEC1*, *LEC2*, *FUS3*, and seed storage genes in seedlings since 4 days after imbibition, the magnitude of which is elevated by sucrose (Tsukagoshi et al., 2007). VAL1 and VAL2 binding and correlation with K27me3 at selected loci (*DOG1*) by ChIP-PCR in seedlings (Chen et al., 2020). **Omics datasets:** ATH1 microarray (see description in the *val1* row in this table) GSE119715 (RNA-seq of 14-day-old *val1*, *val2*, *val1 val2*, and wild-type seedlings). GSE145387 (ChIP-seq of VAL1-GFP and VAL2-GFP in wild-type background, and ChIP-seq of H3K27me3 of *val1 val2* and wild-type samples using 14-day-old seedlings). GSE159499 (ChIP-seq of SWN-GFP in *val1 val2* or wild-type background using 14-day-old seedlings) (Yuan et al., 2021).
*vil1* (*vrn5*)	**Dormancy and germination:** Hypersensitive to ABA, delayed germination and cotyledon greening, and reduced root length on ABA plates (Zong et al., 2022).	**Selected marker genes:** VIL1 binds to *ABI3*, *ABI4*, and *RD29*. In the *vil1* mutant, these genes show upregulated transcript abundance and reduced H3K27me3 (Zong et al., 2022). **Omics datasets:** GSE180587 (RNA-seq of 1-day-old *vil1* and Col-0 seedlings) shows that DE genes are enriched for genes related to ABA responses and seed germination programs (Zong et al., 2022); PRJNA973989 (ChIP-seq of VELs before and after vernalization) (Franco-Echevarría et al., 2023).
*vil1 swn*		**Selected marker genes:** Further upregulation of *ABI3* and *ABI4* compared to vil1 single mutant (Zong et al., 2022).
*pkl*	**Dormancy and germination:** *pkl* mutant is hypersensitive to ABA, and shows delayed germination and cotyledon greening on ABA plates (Perruc et al., 2007). **Storage compounds:** Low penetrance of storage lipids and proteins at the root tip, and the trait is enhanced by GA biosynthesis inhibitors (Ogas et al., 1997). **Callus and SE:** Embryogenic callus formed from explants of pickle roots, cotyledons, and hypocotyls from *pkl* plants (Ogas et al., 1997; Henderson et al., 2004).	**Selected marker genes:** Increased and prolonged accumulation of *ABI3* transcript and proteins in imbibed *pkl* seeds and young seedlings (Perruc et al., 2007). Elevated master TF expression in seeds (*FUS3* and *ABI3*) and seedlings (*LAFL*) (Shen et al., 2015). **Omics datasets:** GSE103361 (see description in the *clf* row in this table) GSE186152 (RNA-seq of 14-day-old wild-type and *pkl* seedlings), GSE186156 (RNA-seq of 14-day-old wild-type and *val1 val2 pkl* seedlings), and GSE186157 (ChIP-seq of PKL in 14-day-old wild-type and *val1 val2* seedlings) (Liang et al., 2022).
*pkl pkr2*	**Storage compounds:** *pkr2*. but not *pkr1*, increases penetrance of *pkl* root phenotype (Aichinger et al., 2009).	**Selected marker genes:** Enhanced/synergistic de-repression of *LEC1, FUS3*, and *ABI3* in *pkl pkr2* (no *LAFL* derepression in *pkl2*), but no PKL association/binding in CHIP; decreased H3K27me3 at *LEC1* and *ABI3*, but not *FUS3* (Aichinger et al., 2009). **Omics datasets:** E-MEXP-2140 (microarray of root tips of 5-day-old *pkl*, *pkr2*, *pkl pkr2*, and wild-type seedlings) (Aichinger et al., 2009).
*hda19*		**Selected marker genes:** *LEC1*, *LEC2*, *ABI3*, and multiple seed storage genes, but not *FUS3* are derepressed in 14-day-old *hda19* seedlings; increased active histone marks and reduced repressive histone marks at the derepressed loci. Binding of HDA19 to *LEC1* and *LEC2* (Zhou et al., 2013). Increased levels of H3ac, H4ac, and H3K4me3 active marks, and decreased level of H3K9me2 and H3K27me3 repressive marks in *7S1*, *OLE1*, *ABI3*, *CRA1*, and *LEC2* in *hda19–1*. (Zhou et al., 2020). **Omics datasets:** Fourteen-day-old wild-type and hda19 seedlings were profiled by ChIP-seq for H3K14ac and H3K9me2 (Zhou et al., 2013). GSE166090 (RNA-seq and H3ac ChIP-seq of 14-day-old *hda19, hda6*, and wild-type seedlings) (Feng et al., 2021).
*hda6 hda19*	**Dormancy and germination:** Post-germinative growth arrest (no cotyledon expansion or greening) in ~70% *hda6*/*hda19*:RNAi double repression seedlings (Tanaka et al., 2008). *hda6* RNAi and *axe5–1* (*hda6* splice mutant) are hypersensitive to ABA and salt stress (Chen et al., 2010b). Delayed germination and seedling growth arrest induced by HDAC inhibitor Trichostatin A (TSA) is not affected by GA (Tanaka et al., 2008). **Storage compounds:** Increased accumulation of storage compounds inferred from upregulation of *LEC1, ABI3*, and *FUS3* and upregulation of seed storage genes in TSA-treated seedlings that exhibit similar phenotypes to hda6/19:RNAi (Tanaka et al., 2008). **Callus and SE:** Embryo-like structures on *hda6/19* RNAi shoot (Tanaka et al., 2008).	**Selected marker genes:** *LEC1, FUS3*, and *ABI3* de-repression in *hda6/1*9 RNAI (Tanaka et al., 2008). *CRA/B/C* are derepressed in TSA-induced somatic embryos (Tanaka et al., 2008). *CRA1, OLE1, 2S2*, and *7S1* are derepressed in *hda19–1* seedlings (Zhou et al., 2020).
*hda19 val2*	**Dormancy and germination:** Embryo lethal (Zhou et al., 2013).	
*sdr4l (sfl1/odr1)*	**Dormancy and germination:** Delayed germination of mature seeds (Cao et al., 2020; Liu et al., 2020; Wu et al., 2022; Zheng et al., 2022). **Storage compounds:** Increased accumulation of storage lipids (Wu et al., 2022; Zheng et al., 2022).	**Selected marker genes:** *ABI3*, *FUS3*, and *DOG1* are upregulated in 15-DAP *Atsdr4l-1* seeds (Zheng et al., 2022). *LAFL* and *DOG1* are derepressed in 4-day-old *Atsdr4l-3* and *Atsdr4l-4* seedlings (Wu et al., 2022). *DOG1* is upregulated in maturing *Atsdr4l-1* and *Atsdr4l-2* seeds at 12–18 DAP (Cao et al., 2020). ABA biosynthesis genes *NCED6* and *NCED9* have elevated expression in *Atsdr4l-2* seeds harvested at fresh (Liu et al., 2020). **Omics datasets:** PRJNA663767 (RNA-seq of 4-day-old *Atsdr4l-3, Atsdr4l-4*, and Col-0 seedlings); GSE185388 (ChIP-seq of 4-day-old estradiol induced AtSDR4L seedlings) (Wu et al., 2022). GSE246997 (ChIP-seq of AtSDR4L expressed from its native promoter in ABA-treated 1-DAI seedlings, and H3K27me3 in 1-DAI and 3-DAI Col-0, *Atsdr4l-4*, *Atsdr4l-5* seedlings grown with 1% sucrose) (Lu et al., 2024).
*sfl1 sfl4* (*sdr4l dig2*)	**Dormancy and germination:** Severely reduced germination (Zheng et al., 2022). **Storage compounds:** Increased accumulation of storage lipids (Zheng et al., 2022).	**Selected marker genes:** *LAFL* genes exhibit dynamic expression over the course of seed maturation in *Atsdr4l dig2* mutant: *LEC1* and *LEC2* have comparable expression levels between the double mutant and wild type from 9 to 18 DAP; *ABI3* expression is downregulated in 12-DAP, but upregulated in 15- and 18-DAP mutants seeds; *FUS3* exhibits lower expression than wildtype in 9-DAP, but higher in 12-, 15-, and 18-DAP mutant seeds. *DOG1* is downregulated in the mutant at 12 DAP, and upregulated at 15 and 18 DAP (Zheng et al., 2022).

Loss-of-function mutants in PRC1 and PRC2 components mimic *LAFL* OE phenotypes, supporting a role for PRCs in terminating the embryonic program during seed-to-seedling transition. **Table 1** and **Figure 1** summarize the phenotypes of these mutants and dissect the regulation by the general epigenetic machinery (e.g. core proteins in the PRCs and TrxG) that affects all stages of plant development, as well as developmental stage-specific epigenetic regulators, including the facultative accessory protein of PRCs. Although there is extensive overlap in mutant phenotypes, some epigenetic mutants only show a subset of phenotypes and with variable penetrance. This is because some PRC2 complex subunits are present in all complexes, e.g., FIE and MSI, while others are specific for some developmental stages (e.g., EMF2, VRN2, FIS2, and MEA), and/or their mutant phenotypes are so severe (embryo lethality) that their roles in other developmental stages can be difficult to uncover (FIE, FIS2, and MSI1). For instance, double mutants lacking PRC2 paralogs that are involved in vegetative development and flowering (*swn clf* and *emf2 vrn2*), show delayed germination (*swn clf*) and develop embryonic callus (*swn clf* and *emf2 vrn2*). FIE is a single subunit required in all PRC2 complexes and *fie* mutants are not viable, due to endosperm over-proliferation and embryo arrest at the heart stage. Similar phenotypes are shown by *prc2* mutants, *fis2*, *mea*, and *msi1* (Meinke, 2020). However, evaluation of embryonically-rescued *fie* plants allowed for the discovery of its role in seed-to-seedling transition. Indeed, embryonically rescued *fie* lines displayed delayed germination, somatic embryos, and embryonic callus-like structures. Embryonic callus also develops in double mutants lacking PRC1 paralogs (*atbmi1a atbmi1b* and *atring1a atring1b*), as well as accessory proteins that promote histone deacetylation (*hda6 hda19 RNAi*) or recruit PRCs (*val1 val2*), albeit with different frequencies. The penetrance of the embryonic callus phenotype can be further increased in mutants simultaneously lacking core subunits of PRC1 and PRC2 (*atring1a atring1b clf*) or in the absence of PRC2 and chromatin remodeling factors (*swn clf pkl*) (**Table 1**). Thus, a stable shutdown of *LAFL* and the embryonic program during vegetative development requires a suite of epigenetic regulators.

Interestingly, mutants in accessory proteins such as *al6 al7*, *lhp1*, and *vil1* affect germination, dormancy, and sensitivity to ABA and stress, and show repression of late-acting *LAFL* such as *ABI3*, suggesting more stage- and context-specific roles for these accessory proteins. While TrxG and PcG play opposite roles in regulating flowering time through FLC (Pien et al., 2008), TrxG’s synergistic role with PRC in transcriptional repression during seed-to-seedling transition does not conform to the norm of TrxG in exerting transcriptional activation. The TrxG mutants *atx* and *ult* strongly enhance the phenotype of *emf1*, with *emf1 atx ult* showing swollen roots and embryo- and callus-like structures (Xu et al., 2018). Similarly, the TrxG homolog SDG8 acts synergistically with PRC2 EMF2 in repressing seed maturation genes, as shown by the development of somatic embryos in *emf2 sdg8* (**Table 1**). Altogether, *LAFL* and downstream seed maturation genes serve as excellent models for comprehending the repression of the seed maturation program during the transition from seed to seedling, similar to *FLC* for the transition from vegetative to reproductive phases (Whittaker and Dean, 2017).

### Omics studies facilitate a comprehensive understanding of seed-to-seedling transition

Mutant phenotypes and marker genes are powerful tools to associate regulation with a specific developmental stage. These tools together with omics studies allow for the efficient and comprehensive characterization of biological processes. Transcriptomic and epigenomic datasets confirmed the observation that general epigenetic machinery participates in all major development transitions, and their regulatory specificity is often determined by accessory proteins and interacting transcription factors (Merini et al., 2017; Xiao et al., 2017b). Starting from profiling canonical histone marks such as H3K27me3 (**Table 1**), our understanding of the epigenetic regulation of plant phase transition has been substantially advanced through the integration of genetic, biochemical, cell biology, and multi-omic results. Here, we discuss some representative examples of how transcriptomic and epigenomic data enable a quantitative assessment of regulatory specificity, suggest interactions between regulators, and uncover crosstalk between regulatory machinery.

#### Genome-wide comparisons reveal regulatory specificity of PRC components

Both PRC1 and PRC2 contribute to the repression of the embryonic and seed maturation programs (**Table 1**). Omics studies have strengthened the observation originally made based on marker genes that PRC1 and PRC2 have both shared and unique functions (Wang et al., 2016; Zhou et al., 2017). Furthermore, multi-omic profiling, including chromatin accessibility, H2AK121ub, H3K27me3, and transcriptome of mutants of core and accessory components of PRC1 and PRC2 and wild-type seedlings have shown that PRC1 regulates chromatin accessibility (Yin et al., 2021), and defined PRC1-dependent and -independent repression by H3K27me3 (Kralemann et al., 2020; Yin et al., 2021).

Genome-wide profiles also aid in the characterization of the accessory proteins, which are crucial for general machinery such as PRCs to exert specific roles in plant development and stress responses. The association of LHP1 with PRC2 was supported by extensive overlap between H3K27me3 and genome-wide binding of LHP1 (Turck et al., 2007; Zhang et al., 2007). Additionally, omics assays help to identify or rule out regulators at specific developmental stages. Cross-comparison of differentially expressed (DE) genes and H3K27me3 in the mutants of *lhp1* and PRC core components showed that LHP1 regulates vegetative-to-reproductive transition, but lacks a major role in seed-to-seedling transition (Wang et al., 2016). Furthermore, the accumulation of H2AK121ub is similar between *lhp*1 and wild-type seedlings (Zhou et al., 2017). Collectively, despite the physical association of LHP1 with both PRC1 (Xu and Shen, 2008) and PRC2 (Hecker et al., 2015), the binding of LHP1 to the dormancy promoting loci, ABI3 and DELAY OF GERMINATION 1 (DOG1) (Molitor et al., 2014), and the modest upregulation of DOG1 in lhp1 mutant (Chen et al., 2020) showed a connection to PRC1 and a minor role in germination, with genome-wide evidence indicating that LHP1 mainly functions after the seed-to-seedling transition as a PRC2 accessory protein.

#### Genome-wide comparisons support functional redundancy and protein–protein interactions

As discussed above, the epigenetic machinery functions through multi-protein complexes. ChIP-seq has been widely used to examine target sites of epigenetic regulators. The sheer number of binding sites across the genome provides numerous data points to assess binding similarities of epigenetic regulators and infer functional redundancy and/or protein–protein interactions (PPIs). In the case of PRC core proteins CLF and SWN, the nearly identical binding patterns (Shu et al., 2019), synergistic phenotype of the *clf swn* double mutant (Chanvivattana et al., 2004), and the absence of data showing their physical interaction indicate that these two methyltransferases function redundantly in different variants of PRC2 core complexes. By contrast, genomic data can also be used as supporting evidence in functional characterization of VAL proteins. Both VAL1/HSI2 and VAL2/HSL1 bind to the RY motif (CATGCA/TGCATG), and they homo- and heterodimerize via the PHD-L domain (Chen et al., 2020). The physical interaction and association with the same CRE are further supported by the extensive overlap of VAL1 and VAL2 across the genome (Yuan et al., 2021). Similarly, the physical interaction of the VAL proteins with the PRC2 core components SWN and CLF and with the chromatin remodeler PKL was demonstrated by both PPI assays and genome-wide binding similarities (Yuan et al., 2021; Liang et al., 2022).

#### Genome-wide comparisons suggest crosstalk of epigenetic machinery

The seedling is arguably one of the most vulnerable stages of a plant’s life cycle. Germinating seeds must strike a balance between preserving limited resources to survive uncertain weather patterns in spring and fall and the rapid consumption of storage compounds to establish themselves and outcompete nearby seedlings. To cope with these two seemingly conflicting priorities, master TFs of seed maturation such as *ABI3* and *FUS3* remain inducible by abiotic stresses during the early stages of germination, while these TFs and the seed maturation program are robustly repressed within a few days after germination if environment is favorable. The robust repression requires coordination of various regulators. For instance, the repression involves a transient increase of histone deacetylase activity soon after germination (Tai et al., 2005), reduced accumulation of active histone marks and increased accumulation of repressive marks at seed maturation and dormancy loci within the first 3 days of germination (Yang et al., 2022a; Pan et al., 2023a), and participation of histone variants (Zhao et al., 2022b). Consistent with the multifaceted regulation, higher-order mutants defective in multiple epigenetic machinery often exhibit more severe phenotypes in germination and seedling establishment (**Table 1**). The coordination of gene repression is often facilitated by PPI. For instance, VAL1 serves an interaction hub to unite the HDAC and PRC activities (Baile et al., 2021; Mikulski et al., 2022), presumably to reduce the level of active marks such as H3ac, enhance the level of repressive histone marks, and limit chromatin accessibility through H2AK121ub and H3K27me3 (Mikulski et al., 2022). Besides VAL proteins, other TFs that possess an EAR motif can also recruit HDAC and enhance H3K27me3 marking through their physical interaction with TPL or SAP18 (Baile et al., 2021). Crosstalk between repressive machinery has been revealed by omics assays. For instance, LHP1 interacts with ATRX, a chromatin remodeler that deposits histone variant H3.3. The intersection of LHP1 target genes with DE genes in *atrx* mutant connects repressive histone marks with histone variants (Wang et al., 2018). Another example is the potential crosstalk between PRC and constitutive heterochromatin in the pericentromeric regions. NDX was discovered as a PRC1-associated protein that regulates ABA sensitivity (Zhu et al., 2020). Recently, genome-wide profiling revealed that NDX binds to heterochromatic small RNA loci and affects non-CG DNA methylation (Karányi et al., 2022), suggesting a potential connection between PRC1 with constitutive heterochromatin and chromatin topology.

## A new puzzle piece: *Sdr4* family in model and crop species

AtSDR4L/ODR1/SFL1 is a nuclear-localized, plant-specific transcriptional corepressor that is devoid of known DNA-binding domains (Moon et al., 2016; Subburaj et al., 2016; Cao et al., 2020; Liu et al., 2020; Wu et al., 2022). Here, we review the functional characterization of AtSDR4L based on the features summarized in **Table 1**, **Figure 1**, and omics tools discussed in the previous section.

### AtSDR4L and its paralogs are novel corepressors in Arabidopsis seed-to-seedling transition

The role of AtSDR4L is specific to the seed-to-seedling transition, and its expression increases during seed maturation, peaks in dry seeds, and decreases upon imbibition, subsequent to the expression patterns of *LAFL* that primarily span from embryogenesis to seed maturation (Stone et al., 2001; Baumbusch, 2003; Cao et al., 2020). Loss-of-function mutants of *Atsdr4l* share many phenotypic and molecular characteristics with mutants listed in **Table 1**. Mature seeds of *Atsdr4l* are more dormant (Cao et al., 2020; Liu et al., 2020; Wu et al., 2022; Zheng et al., 2022). *Atsdr4l* seedlings exhibit stunted growth, with seed storage compounds accumulating to various degrees depending on exogenous sucrose and the duration of after-ripening and cold stratification (Wu et al., 2022; Zheng et al., 2022). A large number of seed maturation genes are upregulated in *Atsdr4l* seedlings, and AtSDR4L binds to the upstream region of a subset of these, including *LEC1* and *ABI3* (Wu et al., 2022). Furthermore, AtSDR4L physically interacts with VAL2, and H3K27me3 at a distal regulatory region upstream of *ABI3* is decreased in 3-day-old *Atsdr4l* seedlings (Lu et al., 2024). The lack of strong dedifferentiation phenotypes, such as those shown in *LEC1* and *LEC2* OE and mutants of PRC core components, indicates that not all genes required for the formation of callus-like structures and somatic embryos are misregulated. *Atsdr4l* seedlings resemble *ML1:FUS3*, suggesting that *FUS3* could be an indirect target activated by elevated *ABI3*. Collectively, these data suggest that AtSDR4L functions together with VAL2 to recruit PRC2 to directly or indirectly repress *LAFL* and other seed maturation genes.

A recent study suggested that AtSDR4L shares partial functional redundancies with its paralogs to form a repressive module in Arabidopsis (Zheng et al., 2022). The paralogs are collectively known as Dynamic Influencer of Gene expression (DIGs) and DIG-like (DILs)/ABA-inducible transcriptional repressors (AITRs)/Seed dormancy Four-Like (SFLs) (Song et al., 2016; Tian et al., 2017; Zheng et al., 2022). Similar to AtSDR4L, DIGs and DILs are nuclear localized (Song et al., 2016; Tian et al., 2017) and physically interact with VAL2 (Lu et al., 2024). An *sfl1 sfl4* (*Atsdr4l dig2*) double mutant shows strongly enhanced seed dormancy and embryonic traits in seedlings compared to *Atsdr4l*, suggesting synergy between AtSDR4L and its paralogs (Zheng et al., 2022). On the other hand, *sfl2 sfl3 sfl4* (*aitr2 aitr6 aitr5*, *dig1 dil1 dig2*) triple mutant seeds exhibited reduced dormancy when they were freshly harvested from siliques at 24 days after flowering (DAF), suggesting antagonistic interaction between AtSDR4L and its paralogs at certain developmental stages (Zheng et al., 2022). Additionally, triple mutant seedlings are hyposensitive to ABA and resistant to drought (Tian et al., 2017), while seedlings overexpressing *DIG1* or *DIG2* are hypersensitive to ABA and salt (Song et al., 2016). These lines of evidence suggest that AtSDR4L and its paralogs may have context-specific functions that require further investigation.

### Sdr4 prevents pre-harvest sprouting in rice

**Figure 2:**
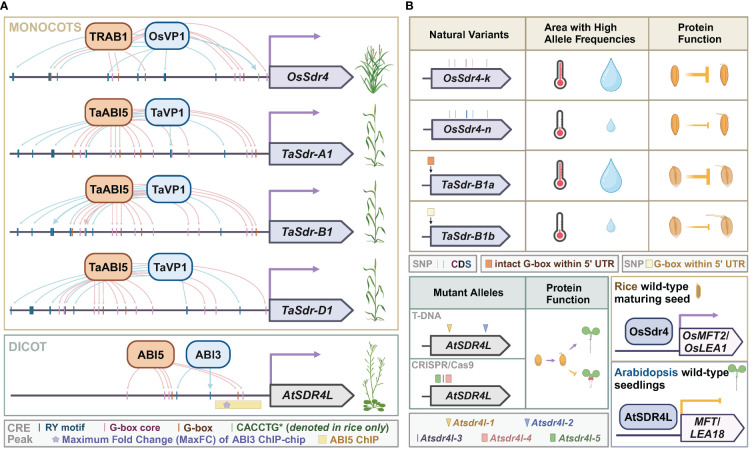
*Sdr4* and its orthologs in monocots and dicots. **(A)** Putative *cis*-regulatory motifs of *OsSdr4* in rice, *TaSdrs* in wheat, and *AtSDR4L* in Arabidopsis from 1 bp to 1,500 bp upstream of the corresponding transcription start site. TF binding sites with experimental evidence are indicated by thick arrows protruding from the regulators of *SDR4*. Data were summarized from O’Malley et al. (2016); Tian et al. (2020); Chen et al. (2021); Wu et al. (2022), and Liu et al. (2024). **(B)** Top: occurrence of natural variants of *OsSdr4* and *TaSdr* in various climates, and their repression of preharvest sprouting. Bottom left: Mutant alleles of *Atsdr4l*, and function of wild-type AtSDR4L in promoting germination and preventing fatty acid over-accumulation. Bottom right: the upregulation of the dormancy-related gene, *MFT*, and the seed reserve-related gene, *OsLEA*, by Sdr4 and the downregulation of these orthologs by AtSDR4L. Data were summarized from Sugimoto et al. (2010); Zhang et al. (2014); Wu et al. (2022), and Zhao et al. (2022a).

*Sdr4* is a major quantitative trait locus and a positive regulator for seed dormancy in rice (Sugimoto et al., 2010; Zhao et al., 2022a). Rice *Sdr4*, herein referred to as *OsSdr4*, is expressed in the embryo and the protein is localized to the nucleus (Sugimoto et al., 2010). *OsSdr4* transcripts begin to accumulate after 7 DAF and increase as seed ripens (Sugimoto et al., 2010). The expression control of *OsSdr4* and its orthologs in wheat, a monocot, and Arabidopsis, a dicot, seems well conserved (**Figure 2A**). Upstream regulatory sequences of *OsSdr4* and orthologs contain multiple RY and G-box (CACGTG) motifs, and binding by ABI3/VP1 and bZIP TFs were shown by *in vitro* or *in vivo* assays in multiple species (O’Malley et al., 2016; Tian et al., 2020; Chen et al., 2021; Wu et al., 2022; Liu et al., 2024). Knocking down or knocking out *OsSdr4* leads to PHS (Sugimoto et al., 2010; Zhao et al., 2022a). In contrast, *Atsdr4l* mutant seeds harvested at maturity exhibit delayed germination (Cao et al., 2020; Liu et al., 2020; Wu et al., 2022). The seemingly opposite mutant phenotypes of seed germination between rice and Arabidopsis may be attributable to the downstream target genes of Sdr4 (**Figure 2B**). In accordance with elevated PHS of *sdr4* mutants in rice, the expression of a gibberellin biosynthesis gene, *OsGA20ox-1*, is significantly upregulated and an ABA-responsive gene, *OsLEA3*, is significantly downregulated compared to wild-type seeds (Sugimoto et al., 2010; Chen et al., 2021; Zhao et al., 2022a). A few other *OsLEA* genes and a dormancy regulator *OsMFT2* are downregulated in the rice *sdr4* mutant as opposed to the upregulation of their orthologs in the *Atsdr4l* mutants (Wu et al., 2022; Zhao et al., 2022a). Interestingly, a recent spatiotemporal single-cell transcriptomic profile of germinating rice embryo revealed that both *OsSdr4* and *OsMFT2* are expressed in the scutellum, and share a similar temporal expression pattern as the transcript abundance of both genes sharply decreases after imbibition (Yao et al., 2024). While mature *Atsdr4l* seeds are more dormant, mutant seeds prematurely harvested at 14 DAF germinate better than wild-type seeds (Zheng et al., 2022), suggesting that temporal-specific regulation may also contribute to the phenotypic difference between rice and Arabidopsis mutants. Storage reserve genes were upregulated in *Atsdr4l* seedlings in Arabidopsis whereas seed storage catabolism genes are upregulated in *Ossdr4* seeds in rice (Wu et al., 2022; Zhao et al., 2022a). The major forms of storage reserves in Arabidopsis are lipids and storage proteins that are deposited in cotyledons, and most of the endosperm except the peripheral endosperm layer in Arabidopsis is consumed by the embryo for nutrient uptake during seed maturation (Sreenivasulu and Wobus, 2013; Doll and Ingram, 2022). In contrast, most of the endosperm in Poaceae is retained and accumulates a substantial amount of starch and storage proteins, followed by programmed cell death without full degradation (Sreenivasulu and Wobus, 2013). Thus, the contrasting role of Sdr4 and its annotated orthologs in grasses and dicots may be associated with opposite regulation of key downstream genes, different types of the major forms of storage compounds, and a higher embryo-to-endosperm ratio in Arabidopsis than that in wheat and rice. In summary, Sdr4 homologs in rice and Arabidopsis share similar expression patterns in seeds and the nuclear localization. However, their function in regulating dormancy is species-, developmental stage-, and tissue-dependent.

### Allelic variations of *Sdr4* correlate with regional weather patterns

Allelic variants of *Sdr4* and its orthologs in coding and regulatory sequences are associated with quantitative differences in seed dormancy, germination, and post-germinative growth. In Arabidopsis, developmental defects include inhibited root growth, swollen hypocotyl, and excess buildup of storage lipids. These defects are more severe in the CRISPR/Cas9 frameshift or segmental deletion mutants of *Atsdr4l-3*, *Atsdr4l-4*, and *Atsdr4l-5* than in the T-DNA insertion mutants of *Atsdr4l-1* and *Atsdr4l-2*, possibly because the mutations reside in the CRISPR/Cas9 lines are in the closer downstream of *AtSDR4L* start codon (Wu et al., 2022; Lu et al., 2024) (**Figure 2B**). In rice, a nearly isogenic line of *OsSdr4* [NIL(*Sdr4*)], in which the genomic segment containing *OsSdr4* from the Kasalath (*indica*) group was introgressed into a Nipponbare (j*aponica*) background, had a substantially lower rate of seed germination than that of Nipponbare, demonstrating that the Kasalath allele of *Sdr4* (*OsSdr4-k*) confers deeper dormancy than the Nipponbare allele (*OsSdr4-n*) (Sugimoto et al., 2010) (**Figure 2B**). The amino acid sequences of *OsSdr4-k* and *OsSdr4-n* alleles differ by approximately 10 amino acid residues, which could potentially affect OsSdr4’s characteristics as a cofactor, thus changing binding behaviors to downstream target loci. The *japonica* group has only the *OsSdr4-n* allele, whereas the *indica* group has both *OsSdr4-k* an*d Sdr4-n*. Analysis of SNPs flanking the *OsSdr4-n* locus in the *indica* cultivars indicated their *OsSdr4-n* allele was introgressed from the *japonica* group. A subsequent larger-scale study revealed a correlation between allele frequency and weather patterns (Zhao et al., 2022a). Allele frequency of *OsSdr4-k* and sequence-similar *OsSdr4-k'* is higher in regions with high annual temperatures and precipitation, whereas *OsSdr4-n* is more prevalent in areas with lower annual temperatures and precipitation. Interestingly, different geographic distribution for *Sdr4* alleles was also reported in wheat (Zhang et al., 2014). Among three homeologs of wheat *Sdr4*, namely, *TaSdr-A1*, *TaSdr-B1*, and *TaSdr-D1*, the *TaSdr-B1b* allele carries an SNP that abolishes a G-box in the 5’ UTR immediately upstream of its start codon and is associated with increased germination compared to that of *TaSdr-B1a* allele with an intact G-box. The mutation in G-box could affect the binding by bZIP and bHLH TFs t *TaSdr-B*. Between the two alleles, *TaSdr-B1a* is likely positively selected for resistance to PHS, since the allele frequency of the *TaSdr-B1a* is high in areas that are more susceptible to severe PHS and low in areas with reduced rainfall and less damage by PHS. These observations in rice and wheat suggest that selection for *Sdr4* alleles best adapted to local climates is a shared feature for the adaptation of staple grains.

## Regulatory symmetries of activators and repressors at the same CREs for developmental transitions

### Regulatory symmetry via the RY motif

**Figure 3:**
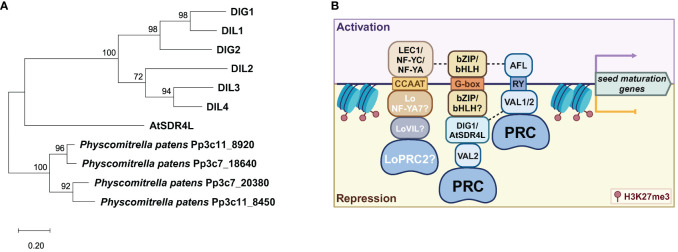
Regulatory symmetry in the activation and repression of seed maturation genes. **(A)** A phylogenetic tree of the AtSDR4L family in Arabidopsis by the maximum-likelihood method and the JTT-matrix-based model using MEGA11 (Tamura et al., 2021). AtSDR4L’s orthologs in *Physcomitrella patens* were used as the outgroup (Lang et al., 2018). **(B)** A working model of the regulatory symmetry between activators and repressors at shared CREs upstream of seed maturation genes. CCAAT is bound by the NF-Y activator complex (top) in Arabidopsis or by the repressor complex of LoNF-YA, LoVIL, and PRC2 in lily (bottom). In Arabidopsis, bZIP/bHLH binding to G-box by itself upregulates the seed maturation genes whereas the G-box binding by bZIP/bHLH coupled with a repressor module of DIG/AtSDR4L-VAL-VIL-PRC2 downregulates these genes. The RY motif can be bound by ABI3/FUS3/LEC1 (AFL) and VAL1/2-PRC, for the activation and repression of the downstream genes, respectively. Dashed lines indicated potentially synergistic effects through protein–protein interaction. Data were summarized from Lumba et al. (2014); Song et al. (2016); Myers and Holt (2018); Bryant et al. (2019); Chen et al. (2020); Liu et al. (2020); Wu et al. (2022), and Pan et al. (2023b).

The regulatory symmetry is the activation and repression of genes through the same CRE. The regulatory summary of the seed maturation programs by B3 proteins is well established (Suzuki and McCarty, 2008). The RY motif is enriched in many seed maturation genes and is often bound by the B3 TFs FUS3, LEC2, and ABI3 for transcriptional activation, as well as VAL1/HSI2 and VAL2/HSL1 for transcriptional repression (Reidt et al., 2000; Nakabayashi et al., 2005; Suzuki et al., 2007; Tsukagoshi et al., 2007; Suzuki and McCarty, 2008; Jia et al., 2014; Yuan et al., 2021) (**Figure 3**). VAL1 and VAL2 can homo- or heterodimerize to target RY motifs in the *DOG1* promoter and repress its expression in seedlings (Chen et al., 2020). However, the B3 domains of these TFs exhibit high similarity but differential binding affinity to the target CREs, with the B3 domain of LEC2 (LEC2-B3) and FUS3-B3 having greater affinity than that of VAL1-B3 and ABI3-B3 (Jia et al., 2021), and with VAL1-B3 binding more effectively than VAL2-B3 (Chen et al., 2020). These varying RY-binding efficacies are likely owing to the slightly different structural bases of the B3 domains and the absence or presence of additional domains (Jia et al., 2021). This difference may also explain the more constrained and specific roles of LEC2 and FUS3 in the establishment and maintenance of the embryonic states, as well as lending greater flexibility for ABI3 to incorporate additional cues into more complex target recognition (Jia et al., 2013, 2021; Roscoe et al., 2015).

### RY and G-box motifs function coordinately in the activation of seed maturation genes

Transcriptional activation of maturation genes *via* the RY motif is often coupled with the G-box motif. G-box motifs are preferentially bound by the basic leucine zipper (bZIP) and basic helix-loop-helix (bHLH) TFs, and these CREs are an overrepresented CRE in seed maturation genes (Nakabayashi et al., 2005; Mönke et al., 2012; Mendes et al., 2013; Ezer et al., 2017; Jo et al., 2020). ABI5, a bZIP TF that interacts with ABI3, was found to transactivate the promoter of *AtEm6* (Nakamura et al., 2001; Lopez-Molina et al., 2002). G-box-mediated transactivation of gene expression by ABI5 is indicated by the numerous downregulated genes in *abi5* dry seed and the enrichment of G-box from these repressed genes (Nakabayashi et al., 2005). Binding to the same RY-containing region upstream of *DOG1*, AFL TFs may also upregulate *DOG1* expression through collaboration with bZIP67 during seed maturation (Bryant et al., 2019). Consistently, G-box and RY motifs are highly enriched in the regulons of ABI3 and FUS3 (Mönke et al., 2012; Wang and Perry, 2013), and ABI3 is believed to induce seed maturation genes via G-box motifs that are in close proximity with RY elements (Suzuki et al., 2003; Jo et al., 2020).

### G-box motif and the repression of the seed maturation program

Loci with high occurrence of RY and G-box elements are frequently associated with high occupancy of the repressive histone mark H3K27me3 in the seedlings (Wang et al., 2016; Liu et al., 2019; Baile et al., 2021). Because both AFL and VAL possess the RY-binding B3 domain, AFL may compete with VAL-PRC for the same RY sites in the regulatory regions of seed maturation genes. Emerging evidence suggests that G-box might be recognized by gene repression machinery (**Figure 3**). Recent studies suggest that AtSDR4L and its paralogs are important for the G-box-dependent transcriptional repression of the seed maturation genes (Song et al., 2016; Liu et al., 2020; Wu et al., 2022). G-box is enriched in AtSDR4L binding regions (Wu et al., 2022). Since AtSDR4L is devoid of known DNA binding domains, it is most likely recruited by bZIP and bHLH family TFs with sequence-specific binding activities to the G-box (Lumba et al., 2014; Liu et al., 2020). It is proposed that AtSDR4L physically interacts with bHLH57 to indirectly inhibit the expression of ABA biosynthetic genes *9-CIS-EPOXYCAROTENOID DIOXYGENASE6* (*NCED6*) and *NCED9*, thereby downregulating ABA biosynthesis to counteract seed dormancy (Liu et al., 2020).

Interestingly, the RY motif (CATGCA) is also found in high frequency in AtSDR4L target sites (Lu et al., 2024). Additionally, *AtSDR4L* and its orthologs also harbor abundant G-box and RY CREs in their own upstream regulatory regions (**Figure 2**). In Arabidopsis, ABI3 binds to the *AtSDR4L* promoter and upregulates its expression in the developing seeds (Wu et al., 2022; Zheng et al., 2022). In turn, AtSDR4L represses *ABI3* to shut down the seed maturation program in the seedlings, promoting the shift to the vegetative phase (Wu et al., 2022). Intriguingly, AtSDR4L also targets itself, possibly through the G-box and RY motifs in its own promoter (Lu et al., 2024). Similarly, the *OsSdr4* promoter contains seven RY and six G-box elements, and one of the RY motifs is in close proximity to a G-box (Sugimoto et al., 2010). The rice ortholog of ABI3, OsVP1, perhaps in collaboration with a rice bZIP TF TRAB1, can induce the expression of *OsSdr4* (Sugimoto et al., 2010; Chen et al., 2021). *OsSdr4* expression substantially decreases in *Osvp1* mutant embryos at the maturation stage. A similar mechanism is conserved in wheat, as TaVP1 binds to the RY motifs, and TaABI5, an ortholog of *Arabidopsis* bZIP TF ABI5, binds to the G-box in *TaSdr* promoter to transcriptionally activate *TaSdr4* (Liu et al., 2024). These conserved regulatory mechanisms on a key locus of dormancy control in both the model plant and crop species provide further elucidation of the mirrored targeting behaviors by activators and repressors.

### Regulatory symmetry in the activation and repression of seed maturation genes *via* CCAAT and GAGA motifs

Symmetry of activator-repressor binding is not limited to the RY and G-box pairing. The CCAAT motif is a CRE frequently found in the promoters of many genes and specifically targeted by the Nuclear Factor Y (NF-Y) factors for gene regulation (Calvenzani et al., 2012). In *Arabidopsis* and soybean, the pioneer TF LEC1 (NF-YB factor) can bind to CCAAT box elements as a trimeric complex with the NF-YA and NF-YC subunits to activate the embryonic programs (Yamamoto et al., 2009; Jo et al., 2020) (**Figure 3**). Conversely, LoNF-YA7 in lily bulbs has been reported to recruit LoVIL1-PRC2 machinery to *LoCALS3* locus at CCAAT motif for H3K27me3 deposition, thereby repressing *LoCALS3* expression to promote the release of bulb dormancy (Pan et al., 2023b). Similarly, GAGA box-binding BPCs can repress *LEC2* during germination (Xiao et al., 2017b) and *FUS3* (Wu et al., 2020) in the ovule integuments and endosperm, but activates *LEC2* in the embryo (Berger et al., 2011). This is in agreement with GAGA-binding proteins in animals, which function as both activators and repressors (Berger and Dubreucq, 2012). Collectively, these results show that transcriptional activation and repression through the same CREs may be a general mechanism in the activation and repression of the seed maturation program. Regulatory robustness and specificity may be determined by functional coordination of transcriptional regulators that binds to these CREs.

## Conclusions and perspectives

### Perspectives and challenges to advance basic knowledge

To date, many players involved in the repression of the seed maturation program during the seed-to-seedling transition have been identified. While existing omics data are tremendously useful to understand the general machinery, they often do not fully capture the dynamics of the regulation in seed germination and seedling establishment because many profiling assays were carried out using 10- to 14-day-old seedlings (**Table 1**). This mismatch with the developmental stages might also miss the identification of stage-specific regulators. For instance, trichostatin A (TSA) treatment to inhibit histone deacetylase activities in 3- and 16-day-old seedlings identified distinct sets of DE genes (Chang and Pikaard, 2005; Tai et al., 2005). Presumably, some regulators of the seed-to-seedling transition might yet to be discovered due to stage-specific regulation and functional redundancy of homologous genes. Additionally, cell biology and biochemical approaches are not always readily applicable to seeds because the seed coat serves as a physical barrier that blocks light and many chemicals. Therefore, tissue- and cell type-specific datasets are often scarce for mature seeds. Previously, transcriptome profiling of dissected developing or germinating seeds (Belmonte et al., 2013; Dekkers et al., 2013) demonstrated tissue- and stage-specific gene expression in Arabidopsis. Single-cell and spatial transcriptomics (Yao et al., 2024) will further advance our understanding of the heterogeneity of gene expression in seeds, allowing a superior statistical power to classify genes based on their expression patterns and designate marker genes to existing and new cell types, thus providing a foundation for understanding cell type-specific GRN. Additional advances may come from integrative analysis of histone modification with omics datasets such as time-course profiles of transcriptome (Narsai et al., 2017), DNA methylation (Bouyer et al., 2017; Kawakatsu et al., 2017), chromatin accessibility and non-coding RNA (Tremblay et al., 2024) during seed-to-seedling transition, and changes in chromatin topology. For instance, histone modifications have been profiled in many epigenetic mutants (**Table 1**). Combined with other assays such as Hi-C (Huang et al., 2021; Yin et al., 2023) and Hi-ChIP (Huang et al., 2021), these datasets help to reveal how histone marks such as H3K27me3 and H2AK121ub impact spatial genome organization by regulating local and long-range chromatin interactions. Collectively, these data elucidate the unique and shared role of PRC1 and PRC2 in the co-regulation of gene expression, and may contribute to a knowledge framework of multi-loci expression optimization and trait stacking for crop improvement.

### Perspectives and challenges for knowledge transfer from model species in the lab to crops in the field

Several PRC-controlled traits, such as dormancy, stress responses, and flowering time, are related to plants’ adaptation to various environments. Regulators specifically targeting these traits are likely to have immediate application value in the field. However, several gaps need to be addressed for knowledge transfer from model species to crop and from controlled laboratory environment to the field. For instance, many important crops and oilseeds are polyploid, which requires additional considerations for homeolog redundancy and subgenome dominance besides optimizing species- and lineage-specific regulation (Ramírez-González et al., 2018; Xiang et al., 2019; Khan et al., 2022). Environmental factors and plant–biotic interactions are prevalent in the field, making trade-offs important considerations to enhance plant performance. For instance, overexpression and mutant phenotypes of master TFs and general epigenetic regulators often reduce fitness, thus requiring more sophisticated engineering of these factors if increased yield under less water and fertilizer usage is the ultimate goal for crop improvement. In summary, research on transcriptional and epigenetic regulation has provided valuable insights into the phase transition from seed to seedlings, and multi-omic studies have revealed many target-specific regulations and crosstalk between regulatory machinery. Further research to identify developmental stage-specific regulators and CREs with minimized fitness trade-off holds strong potential to engineer crops that can adapt to the increasingly stressful environments associated with the increasingly volatile weather patterns from a warming climate.

## Author contributions

DG: Conceptualization, Visualization, Writing – original draft. BL: Writing – original draft. MA: Writing – original draft. SG: Conceptualization, Funding acquisition, Writing – review & editing, Writing – original draft. LS: Conceptualization, Funding acquisition, Supervision, Writing – original draft, Writing – review & editing.

## Glossary

**LEC1, FUS3, LEC2, ABI3 (LAFL)**
LEC1: LEAFY COTYLEDON 1
FUS3: FUSCA 3
LEC2: LEAFY COTYLEDON 2
ABI3: ABSCISIC ACID INSENSITIVE 3
**Polycomb Repressive Complex 1 (PRC1) Core**
AtRING1A, 1B: *Arabidopsis thaliana* RING 1A, 1B
AtBMI1A, 1B, 1C: *Arabidopsis thaliana* BMI1A, 1B, 1C
**Polycomb Repressive Complex 2 (PRC2) Core**
CLF: CURLY LEAF
SWN: SWINGER
MEA: MEDEA
EMF2: EMBRYONIC FLOWER 2
VRN2: VERNALIZATION 2
FIS2: FERTILIZATION INDEPENDENT SEED 2
FIE: FERTILIZATION INDEPENDENT ENDOSPERM
MSI1: MULTICOPY SUPPRESSOR OF IRA 1
**Proteins that Interact with PRC Core**
AL6, 7: ALFIN-LIKE 6, 7
VAL1/HSI2: VIVIPAROUS1/ABI3-LIKE 1/HIGH-LEVEL EXPRESSION OF SUGAR INDUCIBLE GENE 2
VAL2/HSL1: VIVIPAROUS1/ABI3-LIKE 2/HSI2-LIKE 1
VIL1/VRN5: VERNALIZATION INSENSITIVE 3-LIKE 1/VERNALIZATION 5
LHP1/TFL2: LIKE HETEROCHROMATIN PROTEIN 1/TERMINAL FLOWER 2
EMF1: EMBRYONIC FLOWER 1
BPC1, 2: BASIC PENTACYSTEINE 1, 2
AtSDR4L/SFL1/ODR1: *Arabidopsis thaliana* SEED DORMANCY FOUR-LIKE 1/SEED DORMANCY FOUR-LIKE 1/REVERSAL OF RDO5 1
DIG1/SFL2/AITR2: DYNAMIC INFLUENCER OF GENE 1/SEED DORMANCY FOUR-LIKE 2/ABA-INDUCED TRANSCRIPTION REPRESSOR 2
DIL1/SFL3/AITR6: DIG-LIKE 1/SEED DORMANCY FOUR-LIKE 3/ABA-INDUCED TRANSCRIPTION REPRESSOR 6
DIG2/SFL4/AITR5: DYNAMIC INFLUENCER OF GENE 2/SEED DORMANCY FOUR-LIKE 4/ABA-INDUCED TRANSCRIPTION REPRESSOR 5
NDX: NODULIN HOMEOBOX
HDA6, 19: HISTONE DEACETYLASE 6, 19
SAP18: SIN3 ASSOCIATED POLYPEPTIDE 18
**Other Epigenetic Regulators of Seed-to-Seedling Transition**
ATX1: ARABIDOPSIS TRITHORAX 1
ULT1: ULTRAPETALA 1
PKL: PICKLE
PKR2: PICKLE RELATED 2
SDG8: SET DOMAIN GROUP 8
TPL: TOPLESS
TPR1, 2, 3, 4: TOPLESS-RELATED 1, 2, 3, 4
