# Supplementary material for: Voice from both sides: a molecular dialogue between transcriptional activators and repressors in seed-to-seedling transition and crop adaptation
Source: Front Plant Sci. 2024 Aug 6;15:1416216. doi: 10.3389/fpls.2024.1416216 (PMC11333834; doi:10.3389/fpls.2024.1416216)
Supplement: Supplementary file 1 [file Table_1.docx]

# Supplementary Material

## Accessions

| **LAFL** |
| --- |
| AT1G21970 (LEC1), AT3G26790 (FUS3), AT1G28300 (LEC2), AT3G24650 (ABI3) |
| **PcG** |
| PRC1 core  AT5G44280 (AtRING1A), AT1G03770 (AtRING1B), AT2G30580 (AtBMI1A), AT1G06770 (AtBMI1B), AT3G23060 (AtBMI1C)  PRC2 core  AT2G23380 (CLF), AT4G02020 (SWN), AT1G02580 (MEA), AT5G51230 (EMF2), AT4G16845 (VRN2), AT2G35670 (FIS2), AT3G20740 (FIE), AT5G58230 (MSI1), AT2G16780 (MSI2), AT4G35050 (MSI3), AT2G19520 (MSI4), AT4G29730 (MSI5)  PRC accessory / interacting proteins  AT2G02470 (AL6), AT1G14510 (AL7), AT2G30470 (VAL1), AT4G32010 (VAL2), AT3G24440 (VIL1/VRN5), AT5G17690 (LHP1/TFL2), AT5G11530 (EMF1), AT2G01930 (BPC1), AT1G14685 (BPC2), AT1G27461 (AtSDR4L/SFL1/ODR1), AT3G48510 (DIG1/SFL2/AITR2), AT5G63350 (DIL1/SFL3/AITR6), AT5G50360 (DIG2/SFL4/AITR5), AT4G03090 (NDX), AT5G63110 (HDA6), AT4G38130 (HDA19), AT2G45640 (SAP18) |
| **other regulators of seed-to-seedling transition** |
| AT2G31650 (ATX1), AT4G28190 (ULT1), AT2G25170 (PKL), AT4G31900 (PKR2), AT1G77300 (SDG8), AT1G15750 (TPL), AT1G80490 (TPR1), AT3G16830 (TPR2), AT5G27030 (TPR3), AT3G15880 (TPR4), AT4G01460 (bHLH57), AT2G36270 (ABI5) |
| **other marker genes** |
| AT4G27140 (2S1), AT4G27150 (2S2), AT4G27160 (AT2S3), AT4G36700 (7S1), AT5G44120 (CRA1), AT4G25140 (OLE1), AT5G13790 (AGL15), AT5G17430 (BBM), AT2G17950 (WUS), AT3G11260 (WOX5), AT1G62360 (STM), AT4G18960 (AG), AT2G40220 (ABI4), AT1G52890 (ANAC019), AT3G15500 (ANAC055), AT1G18100 (MFT), AT2G35300 (LEA18) |
| **Sdr4 Homologs, regulators, and downstream genes in monocots** |
| Rice:  Os07g0585700 (Sdr4), Os01g0911700 (OsVP1), Os08g0472000 (TRAB1), Os01g0111600 (OsMFT2), Os04g0589800 (OsLEA1)  Wheat:  TraesCS2A02G191400 (TaSDR-A1), TraesCS2B02G215300 (TaSDR-B1), TraesCS2D02G196200 (TaSDR-D1) |
